# Alendronate-Loaded Modified Drug Delivery Lipid Particles Intended for Improved Oral and Topical Administration

**DOI:** 10.3390/molecules21070858

**Published:** 2016-06-29

**Authors:** Lacramioara Ochiuz, Cristian Grigoras, Marcel Popa, Iulian Stoleriu, Corneliu Munteanu, Daniel Timofte, Lenuta Profire, Anca Giorgiana Grigoras

**Affiliations:** 1 Department of Pharmaceutical Technology, Faculty of Pharmacy, Grigore T. Popa University of Medicine and Pharmacy of Iasi, Universitatii Street, 16, Iasi 700115, Romania; 2 Petru Poni Institute of Macromolecular Chemistry, Aleea, Grigore Ghica Voda, 41A, Iasi 700487, Romania; crgrig@icmpp (C.G.); angrig@icmpp.ro (A.G.G.); 3 Department of Natural and Synthetic Polymers, Gheorghe Asachi Technical University of Iasi, Romania, Prof. Dr. Docent Dimitrie Mangeron Avenue, 73, Iasi 700050, Romania; marpopa2001@yahoo.fr; 4 Faculty of Mathematics, Alexandru I. Cuza University, 11 Bvd. Carol I, Iasi 700506, Romania; stoleriu@yahoo.com; 5 Faculty of Mechanical Engineering, Gheorghe Asachi Technical University of Iasi, Romania, Prof. Dr. Docent Dimitrie Mangeron Avenue, 73, Iasi 700050, Romania; cornelmun@yahoo.com; 6 Faculty of Medicine, Grigore T.Popa University of Medicine and Pharmacy Iasi, 16 Universitatii Street, Iasi 700115, Romania; dantimofte@yahoo.com; 7 Surgery Department, Sf. Spiridon Hospital, 1 Piata Independentei, Iasi 700111, Romania; 8 Department of Pharmaceutical Chemistry, Faculty of Pharmacy, Grigore T. Popa University of Medicine and Pharmacy of Iasi, Universitatii Street, 16, Iasi 700115, Romania; nprofire@yahoo.com

**Keywords:** alendronate, Compritol, Gelucire 44/14, Cremophore 25, particles, prolonged release

## Abstract

The present paper focuses on solid lipid particles (SLPs), described in the literature as the most effective lipid drug delivery systems that have been introduced in the last decades, as they actually combine the advantages of polymeric particles, hydrophilic/lipophilic emulsions and liposomes. In the current study, we present our most recent advances in the preparation of alendronate (AL)-loaded SLPs prepared by hot homogenization and ultrasonication using various ratios of a self-emulsifying lipidic mixture of Compritol 888, Gelucire 44/14, and Cremophor A 25. The prepared AL-loaded SLPs were investigated for their physicochemical, morphological and structural characteristics by dynamic light scattering, differential scanning calorimetry, thermogravimetric and powder X-ray diffraction analysis, infrared spectroscopy, optical and scanning electron microscopy. Entrapment efficacy and actual drug content were assessed by a validated HPLC method. In vitro dissolution tests performed in simulated gastro-intestinal fluids and phosphate buffer solution pH 7.4 revealed a prolonged release of AL of 70 h. Additionally, release kinetics analysis showed that both in simulated gastrointestinal fluids and in phosphate buffer solution, AL is released from SLPs based on equal ratios of lipid excipients following zero-order kinetics, which characterizes prolonged-release drug systems.

## 1. Introduction

Sodium alendronate (AL) is a pharmaceutical substance included in class 3 of the biopharmaceutical classification system, with a hydrophilic amphiprotic character and reduced permeability. Currently, AL is administered orally for the therapy of bone diseases such as Paget’s disease, osteoporosis, metastatic bone disease, and malignant hypercalcemia [1,2,3]. AL is used in daily (10 mg/tablet) or weekly (70 mg/tablet with or without 2800 I.U. of vitamin D_3_) conventional release tablets. Tablets with a higher concentration of vitamin D3 (70 mg AL/5600 IU cholecalciferol/tablet) have also been introduced in therapy quite recently [4]. Once-weekly administered tablets (70 mg/tablet) have largely increased patient compliance with AL and led to a reduction in the incidence of gastrointestinal side effects. Pharmacokinetic studies have shown that oral doses of AL in the range 5–80 mg generate a bioavailability of 0.1%–1%, with a fraction of 50% of the amount deposited in the bone. In addition, it was shown that oral administration of 10 mg AL daily and of 70 mg AL once-weekly generates the same level of increase in bone mineral density: 5%–6% in the vertebrae and 3%–9% in the femoral bone. AL administered orally in the form of a solution (70 mg/vial) is intended for patients with deficiencies in swallowing [5,6,7]. The most important disadvantage of AL, however, is its low oral bioavailability (under 1%) caused by several factors such as low permeability due to its negatively charged molecules; short plasma half-time (T_½_ = 0.5–2 h), its chelatation by Ca^2+^ ions resulting in non-absorbable complexes [8]. For these reasons, recent research on AL has been focused on increasing its bioavailability by formulation in modified release pharmaceutical dosage forms intended for oral administration or for other routes of administration (e.g., transdermal, pulmonary, nasal or rectal mucosa) [9,10,11]. The development of new systems of administration and transport is a priority direction of research in increasing the efficiency of osteoporosis medication both in terms of drug bioavailability and of patient compliance [12,13,14]. It has been shown that AL transdermal administration leads to an increase in in vivo availability of AL (10%), while intranasal administration records a 25% increase as compared with oral administration [15,16]. 

Solid lipid nanoparticles (SLPs) are drug delivery systems based on solid lipid matrices stabilized by surfactants [17,18,19]. The present paper focuses on solid lipid particles (SLPs), described in the literature as the most effective lipid drug delivery systems that have been introduced in the last decades [20,21,22], as they actually combine the advantages of polymeric particles, hydrophilic/lipophilic emulsions and liposomes. SLPs exhibit several significant advantages, among which their ability to incorporate both lipophilic and hydrophilic drug substances, excellent biocompatibility and biodegradability, realization of controlled and/or targeted drug release, enhanced bioavailability of loaded compounds, physical-chemical protection of labile active substances, high stability and possibility of preparation by various methods without organic solvents [23,24]. Generally, SLPs enclosing hydrophilic substances are prepared with a high concentration of surfactants, which limits their administration to the oral route [25]. 

The general aim of the present study was to develop AL-loaded SLPs able to increase the bioavailability of AL when administered orally or by external routes. Therefore, we guided our research in two main directions: on the one hand, we focused on the preparation method in order to gain good-quality Al-loaded SLPs suited for various administration routes and, on the other hand, we investigated the in vitro release profile of AL from the prepared SLPs, in various release environments with different pH values which mimic several administration routes. 

In the current study, we present our most recent advances in the preparation of AL-loaded SLPs by hot homogenization and ultrasonication using a self-emulsifying lipidic mixture of Compritol 888 (gliceril behenat), Gelucire 44/14 (a mixture of glycerol and PEG1500 esters of long fatty acids) and Cremophor A 25 (the 25 mole ethoxylate of a blend of cetyl and stearyl alcohols). The AL-loaded SLPs were formulated and investigated for their in vitro drug release kinetics, as well as for their physicochemical and morphological characteristics.

## 2. Materials and Methods

### 2.1. Materials

Alendronate sodium tryhidrate was supplied by Apotex Pharmaceutics Inc. (Weston, FL, USA). Compritol 888 (C 888) and Gelucire 44/14 (G 44/14) were kindly offered by Gatefosse (Nanterre, France). Cremophor A *25* (C 25) was purchased from Sigma Aldrich Co. (Precisa Ltd., Bucharest, Romania). All other reagents and solvents used were of analytical grade. 

### 2.2. Methods

#### 2.2.1. Preparation of Alendronate-Loaded Solid Lipid Particles

AL-loaded SLPs were prepared by hot homogenization followed by utrasonication and lyophilization with various compositions. The self-emulsifying lipid mixtures (Compritol 888, Gelucire 44/14 and Cremophor A 25) were melted by heating at 90 °C on a boiling water bath under continuous stirring. Separately, AL was dissolved in 20 mL of distilled water and heated at the same temperature as the oily phase. The coarse emulsion was prepared by adding the hot aqueous phase to the oily phase dropwise under magnetic stirring at 900 rpm and 90 °C for 15 min. Next, in order to decrease internal particle size, the oil-in-water emulsion was sonicated for 10 min, using a 100 W model UP 100 H ultrasonic processor equipped with a 10 mm Ti sonotrode (Hielscher, Teltow, Germany). The resultant SLPs dispersion was pre-frozen at −20 °C for 12 h, and then the samples were quickly transferred to the alpha 2-4-LSC freeze-dryer (Christ, Osterode am Harz, Germany) at −58 °C for 15 h to obtain the SLPs powders subsequently used in the experiments. 

#### 2.2.2. Characterization of AL-loaded SLPs 

##### SLPs Polydispersity Index and Zeta Potential

SLPs polydispersity index and zeta potential were determined by dynamic light scattering (DLS) using a ZetaNano ZS instrument (Malvern, Worcester, UK) working at 633 nm (incident laser light) and single scattering angle (173°). For this purpose, the lyophilized SLPs were dissolved in Millipore pure water (solution concentrations of 0.05 wt %), allowed to swell for 24 h, and then sonicated in a Branson ultrasonicator (Branson, Danbury, CT, USA) for 30 min. In this way, the homogeneous solutions were prepared for polydispersity index and zeta potential measurements. 

##### Fourier Transform Infrared Spectroscopy (FTIR)

The FTIR spectra were collected on a Vertex 70 spectrometer (Bruker Austria GmbH, Vienna, Austria) using the KBr pellet technique. The KBr pellets were prepared by mixing 3 mg of SLNs sample with 500 mg spectroscopy grade KBr. Spectra were recorded on all eight formulations as well as on raw materials (AL, C25, G 44/14, C888). The spectra obtained were compared with those reported in the literature. 

##### Differential Scanning Calorimetriy (DSC)

DSC was carried out on a Perkin Elmer apparatus (Shelton, CT, USA) under the following conditions: the samples (4–4.5 mg) sealed in an aluminum pan were scanned in the temperature range 50–300 °C in an inert atmosphere (nitrogen flow at 20 mL/min). Prior to the experiment, DSC was calibrated with pure indium as the standard for melting temperature and enthalpy. 

##### Thermogravimetric Analysis (TGA)

TGA measurements were performed on 3–6 mg samples using a Mettler Toledo (Schwerzenbach, Switzerland) derivatograph under a nitrogen atmosphere with a flow rate of 20 mL/min, at a heating speed of 10 °C/min (25–600 °C). 

##### Powder X-ray Diffraction (PXRD) Analysis

PXRD analysis were performed with X’pert Pro MPD/Cubic Fast (Almelo, The Netherlands) by using with a monochromatic Kα radiation (λ = 1.54056 Å) in StepScan mode at a scan rate of 0.035 s^−1^. The anodic current was 40 mA and scanning angle (2θ) was between 10° and 60°.

##### Optical and Scanning Electron Microscopy 

The form and morphological characteristics of SLNs with AL and of the raw materials were studied by optical microscopy in polarized light (OMP) with a Leica stereoscopic microscope (Leica Microsystems, Wetzlar, Germany) in polarized light and by scanning electron microscopy (SEM) on a Vega II SBH microscope (Tescan, Brno, Czech Republic). 

#### 2.2.3. HPLC Analysis and Method Validation

The chromatographic method applied for the assessment of the entrapment efficacy and for the in vitro release tests was developed and validated in-house, according to USP [26]. The determinations were made on a HP 1090 Series II liquid chromatograph (Thermo Fisher Scientific, Waltham, MA, USA) equipped with a multidiode detector, an Agilent 8453 UV-Visible spectrophotometer and a ZORBAX StableBond column SB C18 column (150 × 4.6 mm, 5 µm). The dosing method was validated as follows: the mobile phase consisted of methanol/acetonitrile/water in a ratio of 17.5/17.5/65 at a flow rate of 0.3 mL/min; temperature in the column compartment was kept constant at 30 °C; the volume of injected solution was 20 µL; since alendronate has no UV-visible absorptive functional group, samples were analyzed by a derivatization reaction with fluorenylmethyloxycarbonyl chloride (9-FMOC) under specific reaction conditions. The product of derivatization was detected at λ = 266 nm; linearity was in the range of 2.5 to 80 µg/mL; precision of method varied between 2.5 to 40 µg/mL, calibration curve equation, y = 10.441X + 83.215; the detection limit was 0.4839 µg/mL and the limit of quantification was 1.4621 µg/mL. 

#### 2.2.4. Assessment of Entrapment Efficacy

The quantitative determination of the Al loaded in SLPs was made by dissolving 100 mg SLPs of each formulation studied in 100 mL of methanol under stirring at 600 rpm/10 min. Subsequently, 2 mL of the solution was diluted to 20 mL with the mobile phase. The amount of AL was assessed by HPLC using the validated method described above. The entrapment efficacy of the method expressed as a percentage was calculated according to the following equation:

(1) $$EE=\frac{M_{ALp}}{M_{ALt}}\times 100$$

where: EE—entrapment efficacy of method; M_ALp_—mass of AL experimentally determined in SLPs; M_ALt_—theoretical mass of AL. The determinations were performed in triplicate and the results presented are representative of the mean of the values obtained. 

#### 2.2.5. Study of the in Vitro Dissolution Release 

The in vitro dissolution tests were conducted in three different dissolution media: simulated gastric fluid (SGF), pH 1.2, for two h; simulated intestinal fluid (SIF), pH 6.8, for 70 h; and phosphate buffer, pH 7.4, for 72 h. The test was performed on a SR8 Plus Series (AB & L JASCO, Chatsworth, CA, USA) apparatus 2 (paddles) equipped with a Dissoette autosampler (Hanson Research Corporation, Chatsworth, CA, USA). The experimental protocol was set as follows: a specific amount of AL-loaded SLPs was introduced in vessels containing 500 mL dissolution medium, bath temperature 37 ± 0.5 °C, rotation speed 50 rpm; the sampling interval was set at 30 min during the first 4 h of the test, and at 60 min for the next 68 h, respectively. Aliquots (2 mL) were withdrawn and subjected to HPLC analysis in order to determine the amount of AL released. After every sampling, the aliquots were replaced with equal volumes of medium at 37 °C. All dissolution tests were made in triplicate, with the mean values reported in graphics (relative standard deviation, RSD < 5%). The amount of released AL in the dissolution media was assessed by the HPLC-DAD method described above. 

#### 2.2.6. Analysis of in Vitro Drug Release Kinetics 

In order to predict and correlate the behavior of the in vitro AL release from the SLPs studied, a suitable mathematical model was used. Thus, the experimental data obtained from the in vitro dissolution tests of AL-loaded SLPs were investigated using four predictable models: zero-order and first-order kinetics, Higuchi, and Korsmeyer-Peppas models [27,28,29]. Data fitting was performed by linear and nonlinear regression using Matlab 7.1. Data were presented as mean ± standard deviation and were considered statistically significant at *p* < 0.05. The Akaike information criterion (AIC) and the correlation coefficient R^2^ were the criteria for selecting the model that most dependably describes the release profile of each formula. In a reliable prediction model, the value of R^2^ is as close to 1 as possible, and AIC has the lowest values possible [30,31].

## 3. Results and Discussion 

### 3.1. Preparation of AL-Loaded SLPs

In order to optimize the method of preparation, eight different formulations of AL-loaded SLPs with various compositions were prepared, summarized in Table 1. The AL: solid lipid excipient ratio was maintained constant, while the proportion of surfactants used for emulsification was varied in order to increase the capacity of Compritol 888 to incorporate AL. Gelucire 44/14 is an emulsifying agent well-known for its ability to instantly form an emulsion, while Cremophor A is used as a co-surfactant to potentiate the effect of Gelucire 44/14. Consequently, the proportion of the two surfactants is very important for the quality, size and stability of AL-loaded SLPs. Samples are numbered and labeled F1 to F8 in preparation order.

### 3.2. Characterization of AL-loaded SLPs

#### 3.2.1. SLPs Polydispersity Index and Zeta Potential

DLS was used to assess the size of the AL-loaded SLPs, as well as to obtain valuable information about the degree of dispersity and stability of the prepared particles in solution. The determination of the average value of particle size using DLS is based on the mathematical fluctuation pattern of light scattering by particles in solution derived from the autocorrelation functions of intensity. As observed from the values in Table 2, the size of AL-loaded SLPs varies from 1.04 µm to 3.87 µm, with the lowest particle size in formulation F8.

Polydispersity Index (PdI) is a measure of the width of particle size distribution. PdI values of less than 0.1 are typically referred to as “monodisperse”. PdI calculation results show that formulations F3 and F8 are consistent with a monodisperse suspension, while formulations F1 and F4, containing high amounts of C25, have high PdI values, consistent with high polydispersity values. The zeta potential indicates the degree of repulsion between adjacent, similarly charged particles in dispersion and its value can be related to the stability of colloidal dispersions. For molecules and particles that are small enough, a high zeta potential will confer stability, i.e., the solution or dispersion will resist aggregation. When the potential is low, attraction exceeds repulsion and the dispersion will break and flocculate [32]. Thus, colloids with a high (negative or positive) zeta potential are electrically stabilized while colloids with low zeta potentials tend to coagulate or flocculate. The small negative values of the zeta potential (from −6.0 mV to −13 mV) presented in Table 2 indicate incipient instability (particles tend to aggregate, not to disperse in the solution) due to the fact that attraction forces between charged particles overcome the repulsion forces. Based on the results, we concluded that F8 (where the weight ratio between G44/14 and C25 is 1:1) is the best formulation for the preparation of dispersible AL-loaded SLPs.

#### 3.2.2. Fourier Transform Infrared Spectroscopy

**Figure 1:**
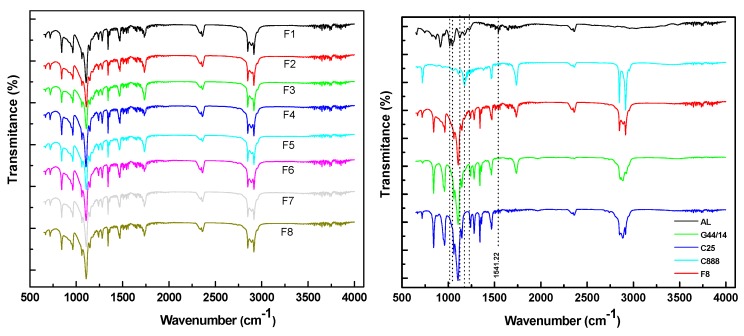
Comparative representation of FTIR spectra recorded for all 8 formulations (**left**) and FTIR spectra of AL-loaded SLPs (formulation F8) and its ingredients (**right**).

The FTIR spectra were recorded on all eight formulations, as well as on AL and excipients, and the results are comparatively presented in Figure 1. By FTIR analysis the characteristic bands of AL were highlighted at 1016.41 (ν_(__P-O)_—asym), 1045.34 (ν_(__P-O)_—asym), 1124.42 (ν_(P=O)_—H bonds), 1174.56 (ν_(P=O)_—H bonds), and 1228.57 cm^−1^ (ν_(__P=O)_—asym) and were related to P-O and P=O bond length stretching. Also, we tracked the intensity change of the peak corresponding to the phosphate group of alendronate (1541.22 cm^−1^) [33]. All these specific bands were marked on Figure 1 (right) with dotted lines. The absence of any significant change and shift in the peaks of the alendronate spectral pattern in the formulations (Figure 1 (right) indicates the absence of any interaction between the drug and the excipients.

The FTIR spectrum recorded on AL-loaded SLPs (F8) represented in Figure 1 (right) resembles a combination of the FTIR spectra of its ingredients. Moreover, as Figure 1 (left) shows, all formulations display similar FTIR patterns. There are slight modifications of the intensity of the transmittance bands from the G 44/14 and C25 compounds. Since the KBr concentration of the sample was kept constant for all FTIR measurements, it is clear that the modification of peak intensities was caused by the proportion variation in the formulations. 

#### 3.2.3 Differential Scanning Calorimetry

**Figure 2:**
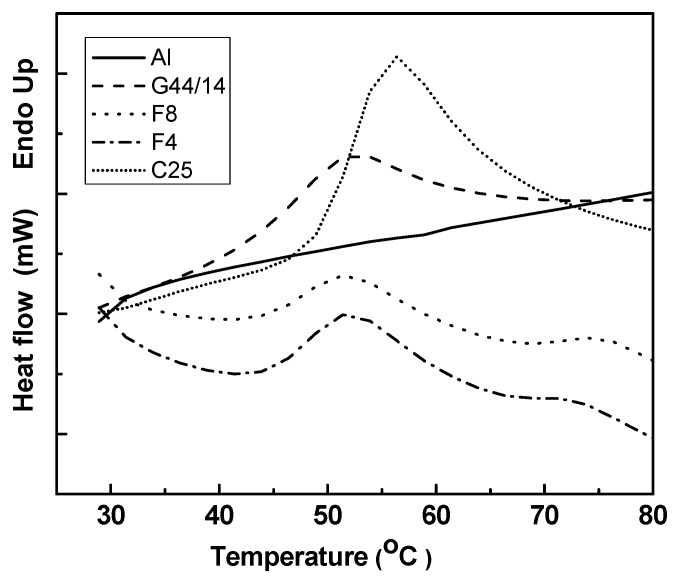
DSC thermograms of AL-loaded SLPs (formulations F4 and F8) and their ingredients.

**Figure 3:**
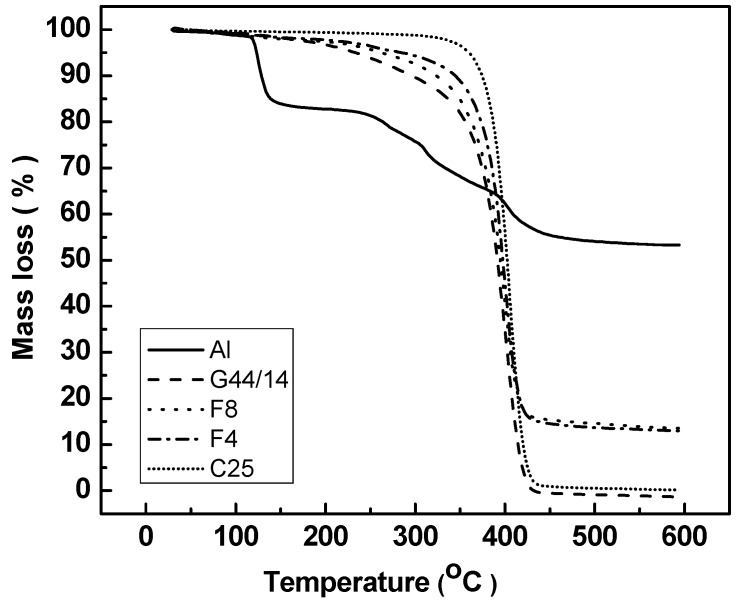
Comparative graphic representation of weight loss for AL-loaded SLPs in F4 and F8 formulations and their ingredients.

DSC is a useful method which reveals the lipid modification process based on the principle that different structures of lipids possess different melting points and melting enthalpies. Lipids possess thermodynamic mobility which attributes for their stable and unstable configuration [34]. In agreement with other research, we consider polymorphism an important route of physical deterioration which affects the stability of a solid system. Though chemically similar, polymorphs exhibit different thermodynamic properties, such as melting point and, besides, the crystalline state is one of the most important parameters affecting drug stability, solubility, dissolution and efficacy [34,35]. In a previous study, we have investigated and demonstrated the stability of C 888 as an agent for forming AL-loaded lipid nanotransport systems [36]. In this study we focused on investigating the compatibility and stability of C25 and G44/14 in combination with AL. Of the studied lipid particle formulations we selected formula F4, which has the highest concentration of C25, and F8, containing equal amounts of the two ingredients. Mention should be made that DSC and TGA analyses were performed for all studied SLPs formulations (F1–F8) and components (AL, C25, C888, G44/14), but due to the low levels of crystalline content of some of them, in this study we chose to present only the relevant results of the samples with higher crystallinity (AL, C25, G44/14, F4 and F8). In Figure 2, DSC results show an extensive melting process in the range of 40–60 °C, which corresponds to the crystalline fraction of PEG moiety present in G44/14 and in F4 and F8. Additionally, AL is stable in this temperature range, as no thermal process was recorded; AL basically melted above 100 °C, while weight loss (as the TG curve shows in Figure 3) and C25 also showed a melting point at 56.4 °C. The DSC analysis shows no difference in the melting points and enthalpies of G 44/14 and F4 and F8 formulation, which is a very important result that confirms the stability of the crystalline forms of fatty acids (G 44/14).

#### 3.2.4. Thermogravimetric Analysis

The weight loss registered (illustrated in Figure 3) indicates an important degradation of AL around 120 °C, simultaneous with its melting. The results confirmed the stability of AL up to 120 °C, but beyond this temperature weight loss is observed at 250 °C and 400 °C. For the rest of the ingredients, a slight weight loss (5%) is observed around 150 °C, and the most important degradation mechanism occurs above 300 °C, when all chemical bonds are broken. 

**Figure 4:**
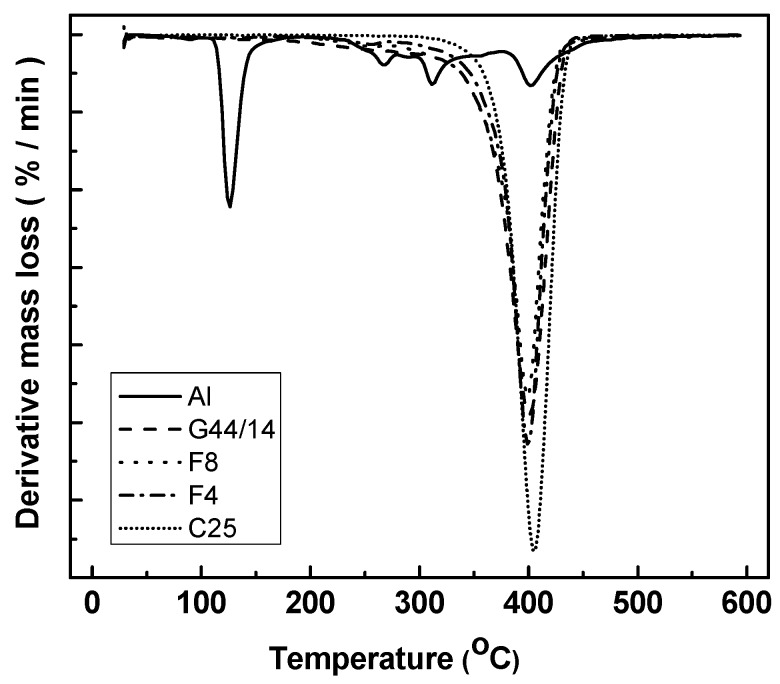
DTG curves for AL-loaded SLPs in F4 and F8 formulations and their ingredients.

DTG signals are consistent with the TG curves, confirming the degradation mechanism of AL which occurs within its melting range at 120 °C (Figure 4). Some decomposition mechanisms leading to the complete degradation of the substance are also observed for AL at temperatures around 270 °C, 310 °C and 400 °C. For all the other ingredients, the main degradation mechanism is observed at around 400 °C with slight differences.

#### 3.2.5. Powder X-ray Diffraction 

**Figure 5:**
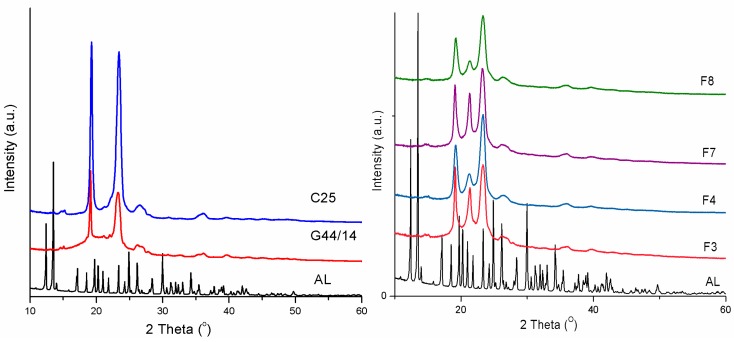
Powder XRD patterns recorded on pure AL, Cremophor, Gelucire (**left**) and formulations F3, F4, F7, F8 (**right**).

Powder X-ray diffraction determines the crystallinity state in lipids, namely the length of long and short spacings of the lipid lattice [37]. This measurement was made on all AL-loaded SLPs. The most relevant XRD patterns presented in Figure 5 show the crystalline state of AL, C 25 and G 44/14 present in formulations F3, F4, F7 and F8. AL, as the active ingredient, showed a series of diffraction peaks, corresponding to the reflections on the planes of the crystalline lattice. AL has a very complex crystalline lattice that remains unmodified in the mixtures. Due to its low amount in the blends, no diffraction peaks characteristic of AL are actually observed in diffractograms, which confirms the very good stability of AL crystals in the formulations. The main diffraction peaks were recorded at 2θ = 19.2° and 2θ = 23.3°, for G 44/14, C 25 and formulations F3, F4, F7, F8. In addition, a new diffraction peak appears around 2θ = 21.4° which is assigned to a small crystalline modification at the interface between C25 and G44/14. The differences in the intensities of these peaks are only related to the variation of the weight fractions of G44/14 and C25 in each tablet. As the DSC results revealed, these new crystalline forms do not affect the thermodynamic stability of the formulations. Additionally, no differences in the 2θ values were recorded in the PXRD patterns of the formulations, which confirms and completes the DSC results, and leads us to conclude that, since no crystalline modification is observed in the formulations, the fatty acid polymorphs are excluded in this case. 

#### 3.2.6. Optical and Scanning Electron Microscopy

**Figure 6:**
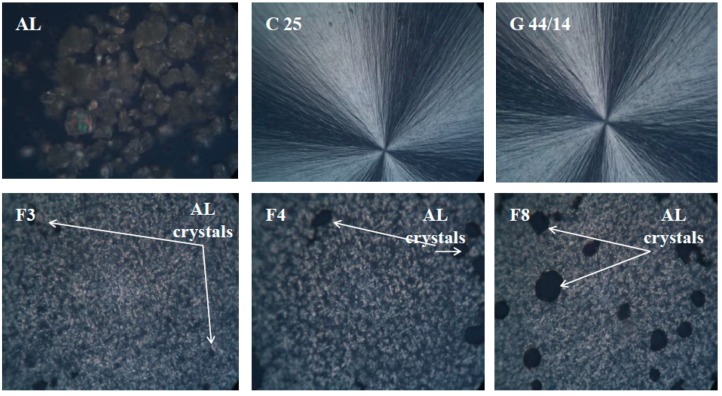
OMP images recorded on pure AL, C 25, G 44/14 and formulations F3, F4, F8.

AL-loaded SLPs and raw materials were analyzed by optical and scanning electron microscopy. Figure 6 shows the most relevant optical micrographs, taken under polarized light of AL, C 25, G 44/14 and formulations F3, F4, F7, F8 at room temperature. The characteristic morphologies for AL, C 25 and G 44/14 can be clearly observed. For formulations F3, F4, F7, F8, the SLPs images show the uniform dispersion of AL crystalline entities among the other ingredients, which results in a homogenous structure. Besides, no phase modification is observed in these formulations. 

**Figure 7:**
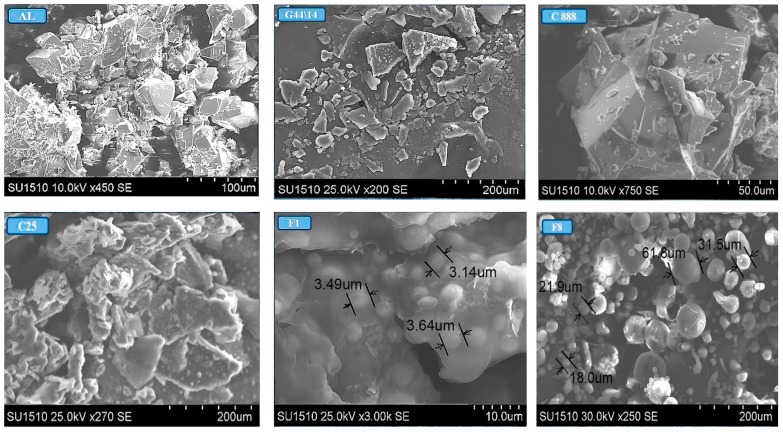
SEM images of raw materials and F1 and F8 AL-loaded SLPs.

SEM confirmed the formation of lipid nanoparticulate systems in the form of spheres with an ordered structure. Figure 7 shows that formulation 8, which contains equal amounts of C 25 and G 44/14, displays free, uniformly dispersed AL-loaded SLPs, while formulation 1, in which C 25 and G 44/14 were in a ratio of 1.7 to 0.7, shows SLPs embedded within a solid mass, possibly of a lipid nature. This may be an indication of the insufficient emulsifying capacity of the two surfactants. 

### 3.3. Entrapment Efficacy

Entrapment efficacy is an extremely important parameter for the evaluation of a method for the preparation of particulate systems. In our study, the efficacy of AL loading in SPLs is in the range 45.6%–65.2%, as shown in Table 2. The highest loading degree was obtained in formulation F7 in which the ratio G 44/14:C 25 was 1.4:1. Significantly, the SLPs formulas with a high content of C 25 generated an AL loading of over 50%, which highlights the important role of this surfactant in the process of emulsification and SLPs yielding*.*

### 3.4. In Vitro Release in Various Dissolution Media

**Figure 8:**
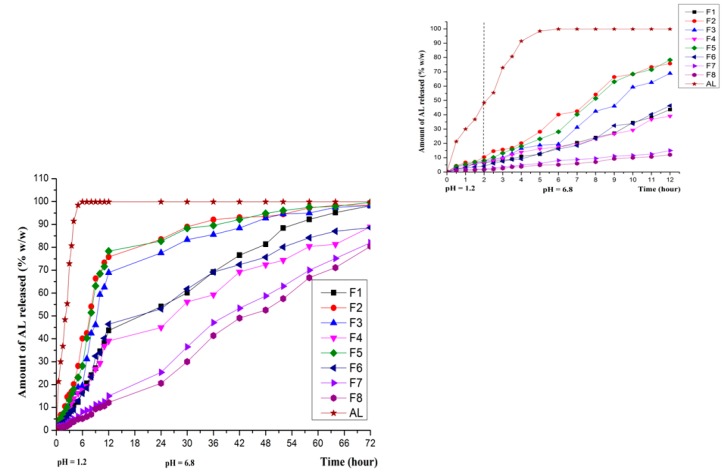
In vitro dissolution release of AL in simulated gastrointestinal fluids (left—72 h; right—the first 12 h).

The in vitro dissolution tests revealed a prolonged release of AL from all eight AL-loaded SLPs formulations compared to the release profile of unencapsulated AL. Results show that in the release medium that simulates the gastrointestinal environment (pH 1.2—simulated gastric fluid medium and pH 6.8—simulated intestinal fluid), F1–F6 formulations generate a ”burst effect” in the first hour of the test (Figure 8), a phenomenon that may be attributed to the modifications of crystallinity recorded by the above-mentioned measurements. However, over 2h, in pH 1.2 dissolution media, F1–F6 SLPs formulations released a low percentage of AL in the range 4.13%–10.43%, while F7 and F8, where the ratio G 44\14 and C25 is equal or almost equal, only released 2.14% and 1.65%, respectively, of the encapsulated AL.It was clearly observed that formulations F7–F8 display a significantly slowed down release profile in the first 12 h of the test, when only 15.01% and 12.11%, respectively, were released of the total amount of AL contained. The release profile recorded during the in vitro dissolution tests is consistent with the formulations with prolonged release [38].

**Figure 9:**
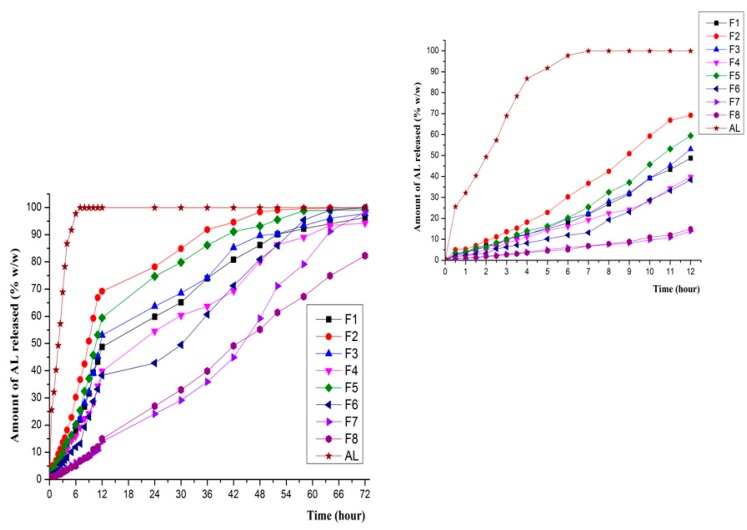
In vitro dissolution release of AL in phosphate buffer pH 7.4 (left—72 h; right—the first 12 h).

A further test, relevant for cutaneous and mucosal administration, was performed in a dissolution medium of phosphate buffer pH 7.4. As in the gastrointestinal simulating media, the slowed-down release of the active principle occurs in this release environment as well (Figure 9). However, the release profile is more rapid in comparison with the first tests realized in the media with pH values of 1.2 and 6.8, a result that may be attributed to the dissociation constant of AL (K_d_) [39]. 

### 3.5. In Vitro Drug Release Kinetics 

The results obtained in the in vitro dissolution tests of AL from the new SLPs formulas were investigated by fitting on four mathematical models specific for modified release dosage forms: zero-order and first-order kinetics, Higuchi, and Korsmeyer-Peppas models.

Zero-order kinetics apply to modified release systems containing less soluble substances. This model defines drug release at a constant rate regardless of the quantity of substance present in the dosage form and it is the perfect model for achieving a prolonged drug substance release [27,40]. A first-order kinetics model is specific to matrix systems in which the active substance release profile varies in direct proportion to the amount of drug remaining inside the dosage form, so that there is a decrease in the amount of drug released over the unit of time [41,42]. Higuchi and Peppas-Korsmeyer models are specific to modified release matrix systems carrying drug release by diffusion [43,44]. Because in the AL-loaded SLPs formulations developed the self-emulsifying mixture of lipid excipients contained various concentrations of G 44/14 (which has in its structure an important hydrophilic part represented by free PEG 1500), we maintain that these models can also be applied to investigate the release kinetics of AL from the new SLPs [45]. The results obtained from data fitting on the four mathematical models are presented in Table 3 for the simulated gastrointestinal fluids and in Table 4 for the phosphate buffer system environment pH 7.4. The evaluation of AL release kinetics from the SLPs emphasized the influence of the self-emulsifying lipid excipients on the profile of the active substance release from the particulate formulations studied. In the gastrointestinal simulation environment, for formulations F7 and F8 that contain almost equal amounts of self-emulsifying lipid excipients, we determined a zero-order kinetics. The other SPLs formulas displayed either a zero-order kinetics or a fitting on the Peppas-Korsmeyer model, as can be seen in Table 3. As regards the analysis of the kinetics of AL release from SLPs in phosphate buffer pH 7 4 medium, for formulas F7 and F8 results revealed a zero-order kinetics as well, while the other six SLPs formulations exhibited a uniform fitting on the first-order kinetic model. The results of the analysis of the kinetics of AL release from SLPs confirm the major influence of the ratio between the self-emulsifying lipid excipients on the characteristics of the active substance release. When the self-emulsifying lipid excipients are used in equal or similar amounts, as is the case of formulations F7 and F8, the SLPs formed are characterized by an osmotic release mainly governed by the structural characteristics of the particulate systems both in simulated gastrointestinal fluids and in phosphate buffer pH 7.4 medium [46,47]. At various concentration proportions between G 44/14 and C 25 we obtained SLPs characterized by a diffusion and erosion release influenced both by the solubility characteristics of the active substance and by the de-structuring properties of the particulate system [48,49].

## 4. Conclusions

The present study highlights the stability and modified and prolonged release of AL from solid lipid particles based on Compritol 888, Gelucire 44/14 and Cremophor 25. The preparation method was optimized in terms of formula composition. The effect of the variation of the ratio between Gelucire and Cremophor was particularly investigated. Based on analyses such as DLS, DSC, TGA, PXRD, OMP and SEM, it was concluded that a ratio of AL:C888:G44/14:C25 of 1:1:1:1 is the optimal composition for the formation of stable, disperse and uniform particles. The size of AL-loaded SLPs prepared with this formula was determined by DLS to be around 1.05 µm. Significantly, the physicochemical tests performed did not reveal important modifications of the structure of the ingredients, although the DSC measurements indicate possible physical interactions between the PEG chains in Cremophor and other components. The method of SLPs preparation has led to a maximum loading efficacy of 65.2% for the ratio AL:C 888:G 44/14:C 25 of 1:1:1.4:1. The in vitro dissolution tests and the analysis of the release kinetics of AL from SLPs have shown that the formulations with an equal, or almost equal, ratio in the mixture of lipid excipients (C 888:G 44/14:C 25 of 1:1.4:1 and C 888:G44/14:C 25 of 1:1:1, respectively), labeled F7 and F8 in this study, yield AL-loaded SLPs which release AL according to a zero-order kinetics. These results indicate that these systems meet the characteristics of modified and extended release delivery systems which may be administered both orally and topically, on mucous membranes or skin. The small gliceride fraction and the free PEG 1500 in the structure of G 44/14 improve AL solubility and wettability in both the simulated gastrointestinal dissolution media and the phosphate buffer pH 7.4. Based on these characteristics, the oral and topical bioavailability of AL-loaded SLPs may be expected to increase. Considering the already proved biocompatibility of raw materials used for the preparation of these AL-loaded SLPs [50,51], we are currently studying these formulations in vivo in order to evaluate the oral and dermal bioavailability of AL in SLPs. The results of this investigation will be presented in an upcoming article.

## Figures and Tables

**Table 1:** Composition of AL-loaded SLPs.

Formulation	Raw Materials (Ratio *w*/*w*)			
	AL	C 888	G 44/14	C 25
**F1**	1	1	0.7	1.7
**F2**	1	1	1.7	0.7
**F3**	1	1	2.4	0
**F4**	1	1	0	2.4
**F5**	1	1	2	0.4
**F6**	1	1	0.4	2
**F7**	1	1	1.4	1
**F8**	1	1	1	1

**Table 2:** DLS characterization and drug content of AL-loaded SLPs.

Formulation	DLS Results			Drug Content	
	Particle Size (µm)	PdI	Zeta Potential (mV)	Actual Drug Content (%) Mean ± SD	Entrapment Efficacy (%) Mean ± SD
**F1**	1.26	1.000	−7.95	13.24 ± 1.25	57.2 ± 2.30
**F2**	2.52	0.231	−10.90	10.22 ± 0.73	50.1 ± 0.99
**F3**	1.13	0.040	−13.10	10.96 ± 1.83	49.2 ± 1.27
**F4**	1.65	1.000	−7.43	14.38 ± 2.52	62.3 ± 1.31
**F5**	3.87	0.364	−11.60	11.39 ± 1.95	45.6 ± 2.78
**F6**	2.40	0.044	−6.43	12.08 ± 3.72	54.9 ± 2.68
**F7**	1.24	0.507	−10.30	14.83 ± 1.19	65.2 ± 0.97
**F8**	1.04	0.030	−13.30	14.56 ± 1.74	60.7 ± 1.16

**Table 3:** Results of curve fitting of the in vitro AL release profile from AL-loaded SLPs in gastrointestinal dissolution media.

Kinetic Model	Model Coefficients	Formula							
		F1	F2	F3	F4	F5	F6	F7	F8
**Zero-order**	**K_0_**	1.6623	1.9536	1.8738	1.4653	1.9490	1.5535	1.1950	1.1075
	**R^2^**	0.8955	0.3726	0.6165	0.8632	0.4447	0.8425	0.9964	0.9950
	**AIC**	120.1776	168.7918	156.6207	118.6883	66.5988	126.9713	25.0111	30.9225
**First-order**	**K_1_**	0.0437	0.0627	0.0559	0.0279	0.0670	0.0313	0.0202	0.0180
	**R^2^**	0.9719	0.9161	0.9597	0.9807	0.9409	0.9790	0.9652	0.9469
	**AIC**	87.3210	118.4923	100.3231	69.7062	110.6022	76.5813	81.8319	90.0754
**Higuchi**	**K_H_**	11.2320	14.1181	12.2087	9.9892	13.9926	10.5979	7.6690	7.0183
	**R^2^**	0.9483	0.8433	0.9031	0.9666	0.8566	0.9420	0.8518	0.8183
	**AIC**	102.587	134.1020	122.2216	83.4667	132.7607	102.0109	118.0376	120.8344
**Korsmeyer-Peppas**	**n**	0.65	0.46	0.49	0.58	0.45	0.62	0.91	0.97
	**K_P_**	6.5280	16.3025	13.6880	7.5003	16.7236	6.6184	1.7005	1.2456
	**R^2^**	0.9823	0.8564	0.9029	0.9852	0.8649	0.9629	0.9955	0.9933
	**AIC**	77.8468	133.9255	124.2792	65.1419	133.2584	92.8413	32.4754	40.3637

**Table 4:** Results of curve fitting of in vitro AL release profile from AL-loaded SLPs in phosphate buffer pH 7.4.

Kinetic Model	Model Coefficients	Formula							
		F1	F2	F3	F4	F5	F6	F7	F8
**Zero-order**	**K_0_**	2.1352	2.6844	2.2234	1.8829	2.4625	1.7802	1.0844	1.1316
	**R^2^**	0.8601	0.6682	0.8580	0.9076	0.8209	0.9365	0.9834	0.9966
	**AIC**	98.5480	124.7318	100.8783	85.3670	110.2489	76.7587	31.7981	0.0578
**First-order**	**K_1_**	0.0395	0.0743	0.0439	0.0311	0.0561	0.0294	0.0144	0.0150
	**R^2^**	0.9803	0.9692	0.9782	0.9874	0.9738	0.9655	0.9509	0.9754
	**AIC**	57.4289	74.8169	61.5543	43.5773	69.8784	63.9492	54.5410	41.4065
**Higuchi**	**K_H_**	11.4848	14.9391	11.9499	10.0080	13.3191	9.2991	5.3645	5.6246
	**R^2^**	0.9167	0.9079	0.9101	0.9088	0.9018	0.8676	0.7579	0.7810
	**AIC**	87.6629	97.8056	91.2823	85.0790	97.6354	92.1918	88.0630	87.3350
**Korsmeyer-Peppas**	**n**	0.65	0.46	0.49	0.58	0.45	0.62	0.91	0.97
	**K_P_**	7.2105	16.8176	12.3038	7.8719	15.3785	6.4837	1.4813	1.2569
	**R^2^**	0.9642	0.8923	0.9036	0.9527	0.8673	0.9379	0.9715	0.9947
	**AIC**	71.9129	103.0962	94.7373	73.2816	105.9575	78.3024	45.1629	11.3721
